# Regioselectivity in the multicomponent reaction of 5-aminopyrazoles, cyclic 1,3-diketones and dimethylformamide dimethylacetal under controlled microwave heating

**DOI:** 10.3762/bjoc.8.3

**Published:** 2012-01-04

**Authors:** Kamal Usef Sadek, Ramadan Ahmed Mekheimer, Tahany Mahmoud Mohamed, Moustafa Sherief Moustafa, Mohamed Hilmy Elnagdi

**Affiliations:** 1 Chemistry Department, Faculty of Science, El-Minia University, El-Minia 61519, Egypt; 2 Department of Chemistry, Faculty of Science for Girls, King Abdul-Aziz University, Jeddah, P.O. Box 50918, Jeddah 21533, Kingdom of Saudi Arabia; 3 Chemistry Department, Faculty of Science, Kuwait University, PO Box 5969, Safat, 13060 Kuwait

**Keywords:** aminopyrazoles, dimedone, DMFDMA, regioselectivity

## Abstract

The multicomponent reaction of 5-aminopyrazole derivatives with cyclic 1,3-dicarbonyl compounds and dimethylformamide dimethylacetal (DMFDMA) in DMF at 150 °C under controlled microwave heating afforded regioselectively 8,9-dihydropyrazolo[1,5-*a*]quinazolin-6(7*H*)-ones **6** rather than the corresponding dihydropyrazolo[5,1-*b*]quinazolin-8(5*H*)-ones **4**.

## Introduction

Several naturally occurring and synthetic compounds containing quinazoline derivatives are of considerable interest in fields related to the organic and medicinal chemistry of natural products [1–2]. The quinazoline ring system represents the core skeleton of an important class of heterocyclic compounds possessing a wide range of biological activities [3–4]. Multicomponent reactions (MCR) occupy an interesting position in organic synthesis because of their atom economy, simple procedures and convergent character [5–7]. An unresolved issue in multicomponent reactions is whether their selectivity is chemo- or regioselectivity, or both, due to the several possible parallel reaction pathways, which result in the formation of different products [8–10]. Many factors modulate the selectivity of synthetic transformations, such as temperature, pressure, solvent, catalyst and type of reaction control, i.e., either kinetic or thermodynamic [11–13]. It has been reported that the use of microwave or ultrasound irradiation provides an additional parameter for synthetic selectivity [14–17].

## Results and Discussion

The multicomponent reaction of 5-aminopyrazoles, dimedone and aromatic aldehydes was reported to afford several different tricyclic products. Thus, in an early report [18], the reaction of the three components in ethanol under conventional heating afforded mainly the corresponding pyrazolo[3,4-*b*]quinolin-5-ones. This finding was later supported by other authors [19]. Recently, the results of an interesting study dealing with such reactions were described by Chebanov et al. [20] Specifically, these researchers performed the reaction at 150 °C in the presence of triethylamine by employing a sealed vessel under microwave or conventional heating, and which thus afforded pyrazoloquinolinones (Hantzsch-type dihydropyridines). On the other hand, the use of sonication at room temperature under neutral conditions favours the formation of isomeric pyrazolo[5,1-*b*]quinazolin-8(4*H*)-ones (Biginelli-type dihydro-pyrimidines) [9]. Employing more nucleophilic bases to catalyse the reaction afforded the corresponding pyrazolo[4,3-*c*]quinazolin-9-ones [20]. It was concluded that, under ambient and neutral conditions, the reaction proceeds under kinetic control, and the Biginelli-type dihydropyrimidines are the predominant isomers. Increasing the reaction temperature in the presence of triethylamine as base produces the more thermodynamically stable dihydropyridine (Hantzsch-type product). In addition, the nature of the catalyst plays an important role [20]. A one-pot three component reaction of 5-amino-1*H*-pyrazole-4-carbonitrile, dimedone and triethylorthoesters in toluene under reflux was recently reported to afford the corresponding pyrazolo[1,5-*a*]-quinazolin-6-one derivatives [21]. Although it is well established that 5-amino-pyrazoles have nonequivalent nucleophilic reaction centres in the aminopyrazole scaffold (N1, C4, NH_2_), which can lead to the formation of several different tricyclic reaction products, no general basis on which to determine the preferred tautomeric form of the final product has been established.

In continuation of our studies in which we performed multicomponent reactions using controlled microwave heating [22–24], we report herein the results of our investigation concerning the regioselectivity in multicomponent reactions of 5-aminopyrazoles, cyclic 1,3-diketones and dimethylformamide dimethylacetal (DMFDMA) under controlled microwave heating.

**Scheme 1:**
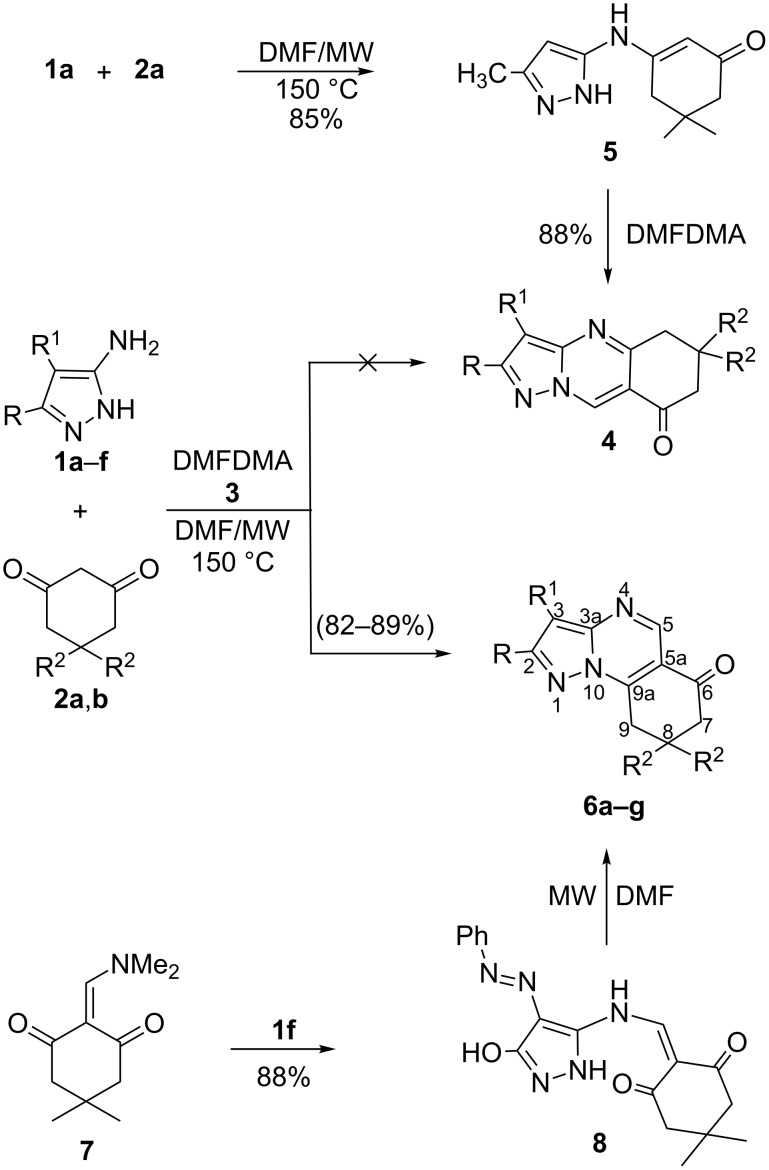
Microwave-assisted synthesis of **4** and **6**.

**Figure 1:**
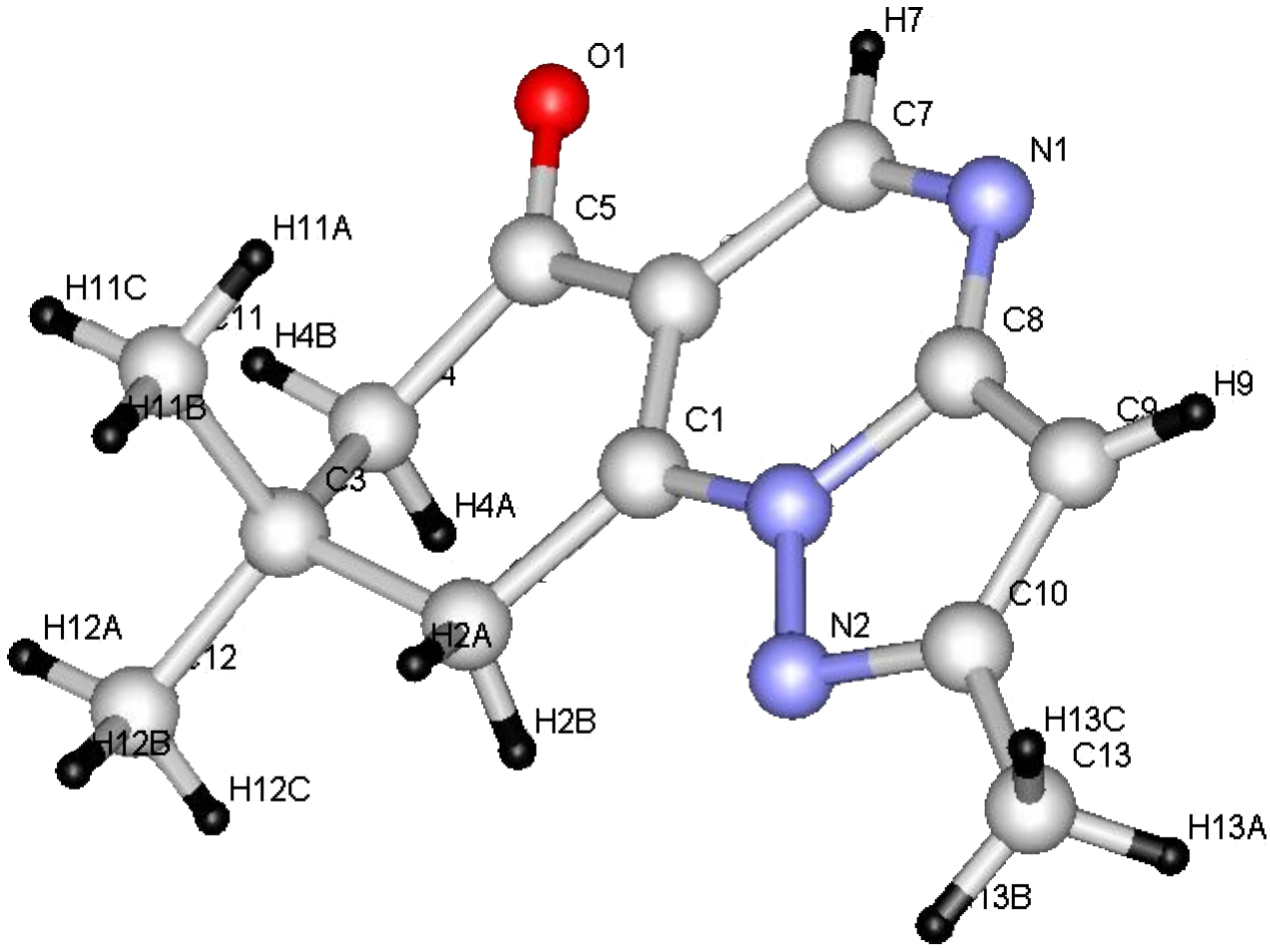
ORTEP diagram of compound **6a**.

**Figure 2:**
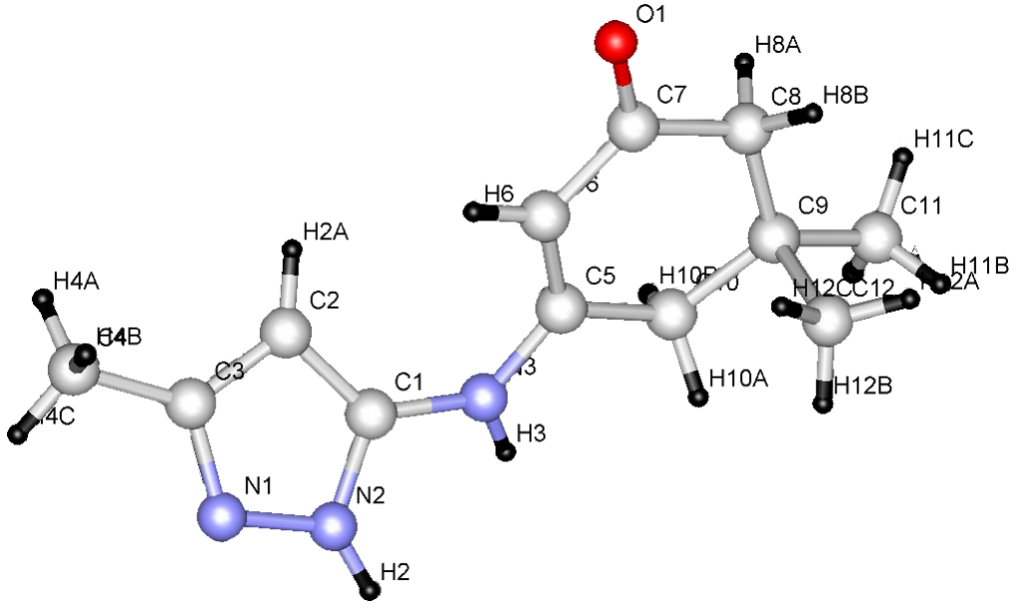
ORTEP diagram of compound **5**.

We began this study by treating 5-amino-3-methylpyrazole (**1a**) and dimedone (**2a**) with DMFDMA (**3**) in DMF under microwave heating at 150 °C for 15 min. After being cooled to room temperature, the precipitated solid product was isolated in 88% yield (Table 1). The mass spectrum of the reaction product showed a molecular ion peak *m*/*z* = 229.12 (100%). The ^1^H NMR revealed a singlet signal at δ = 6.70 ppm integrated for one proton, which was assigned to the pyrazoloquinazolone C_3_ proton, and which indicates the lack of involvement of such a proton in the condensation leading to the tricyclic system. Although, it was previously reported [20] that, due to reduced steric hindrance, the multicomponent reaction of 5-amino-3-methyl-pyrazole, aromatic aldehydes and dimedone under controlled microwave irradiation at 150 °C involves the participation of C_3_-H of the pyrazole ring in such a cyclocondensation reaction, this is not favoured in our case. In addition two signals were assigned to two CH_2_ groups and three methyl functions, and a singlet at δ = 8.75 ppm corresponding to one proton at C_5_. The pyrazolo[1,5-*a*]-quinazolin-8(5*H*)-one **6a** was established as the reaction product, and ^13^C NMR was in agreement with the proposed structure, rather than with isomeric **4a**, which was prepared by first reacting **1a** with dimedone (**2a**) in DMF under microwave heating at 150 °C for 10 min to afford **5**. Subsequently, treating compound **5** with DMFDMA (**3**), under the same experimental conditions, gave compound **6a** in excellent yield (Scheme 1 and Table 1). Furthermore, the structures of compounds **5** and **6a** were unambiguously confirmed by single-crystal X-ray diffraction [25–26] (Figure 1, Figure 2 and Table 1, Table 2, Table 3).

**Table 1:** Microwave-assisted synthesis of **4** and **6**.

entry	compound	5-aminopyrazole, **1**;	cyclic 1,3-diketone, **2**;	product	yield (%)

1	**1a**	R = CH_3_, R^1^ = H	**2a**; R^2^ = CH_3_	**6a**	88
2	**1a**	R = CH_3_, R^1^ = H	**2b**; R^2^ = H	**6b**	85
3	**1b**	R = NH_2_, R^1^ = CO_2_Et	**2b**; R^2^ = H	**6c**	89
4	**1c**	R = CH_3_, R^1^ = C_6_H_5_	**2a**; R^2^ = CH_3_	**6d**	83
5	**1d**	R = C_6_H_5_, R^1^ = H	**2b**; R^2^ = H	**6e**	82
6	**1e**	R = C_6_H_5_, R^1^ = H	**2a**; R^2^ = CH_3_	**6f**	83
7	**1f**	R = OH, R^1^ = C_6_H_5_N=N–	**2a**; R^2^ = CH_3_	**6g**	84

**Table 2:** Selected bond lengths and bond angles for compound **6a**.

bond lengths		bond angles	

atom numbers	geometric parameter (Å)	atom numbers	geometric parameter (°)

N1–C8 N1–C7 N2–C10 N3–C8 N3–C1 N1–C6 N6–C7	1.372 (3) 1.309(3) 1.344 (3) 1.397 (3) 1.490 (3) 1.377 (3) 1.421 (3)	C7–N1–C8 N2–N3–C1 C1–N3–C8 N3–C1–C6 C8–C9–C10 C1–C6–C5 N1–C7–C6 N1–C8–C9 N3–N2–C10 N2–N3–C8 N1–C8–N3	116.15 (19) 125.03 (16) 122.51 (18) 116.10 (17) 106.29 (17) 119.42 (19) 124.5 (3) 133.29 (19) 103.65 (17) 112.41(16) 121.56 (18)

**Table 3:** Selected bond lengths and bond angles for compound **6e**.

bond lengths		bond angles	

atoms numbers	geometric parameter (Å)	atom numbers	geometric parameter (°)

N3–C9 N3–C8 N1–C1 N2–C9 N2–C2 C2–C7 C7–C8	1.360 (3) 1.3147(3) 1.346 (3) 1.396 (3) 1.364 (3) 1.363 (3) 1.428 (3)	C8–N3–C9 N1–N2–C2 N2–C1–C3 N2–C2–C7 C1–C10–C9 C2–C7–C8 N3–C8–C7 N3–C9–C10 N2–N1–C1 N1–N2–C9 N2–C9–N3	116.10 (19) 124.94 (19) 124.71 (18) 116.23 (18) 120.9 (17) 124.7 (2) 105.78 (17) 133.37 (19) 103.94 (14) 112.01(15) 120.99 (18)

With this result in hand, we went on to study the scope of such multicomponent reactions with several substituted 5-aminopyrazoles and cyclic 1,3-diketones. Thus, the reaction of **1b**–**f** with **2a**,**b** and **3**, under the same experimental conditions, afforded the corresponding pyrazolo[5,1-*b*]quinazolin-8(5*H*)-ones **6b**–**g**, respectively. The structures of **6b**–**g** were deduced from their ^1^H NMR, ^13^C NMR, mass spectra and elemental analyses.

**Figure 3:**
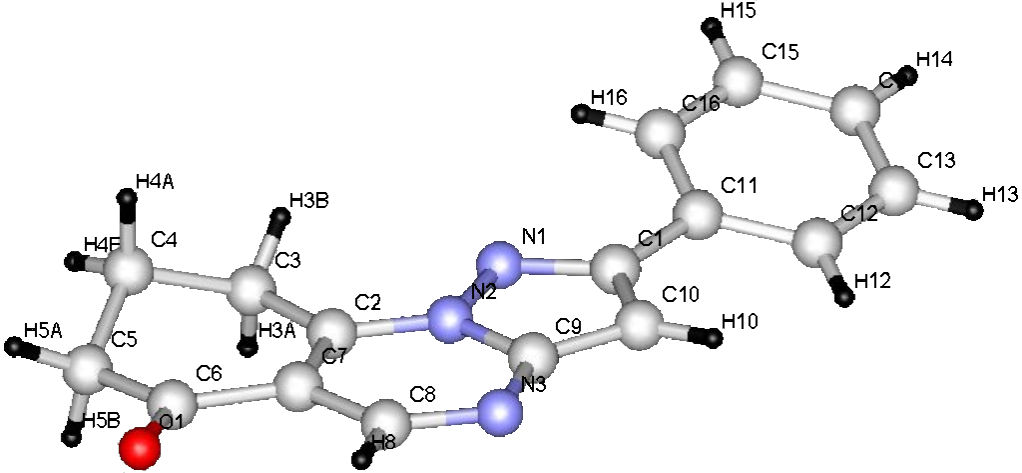
ORTEP diagram of compound **6e**.

**Figure 4:**
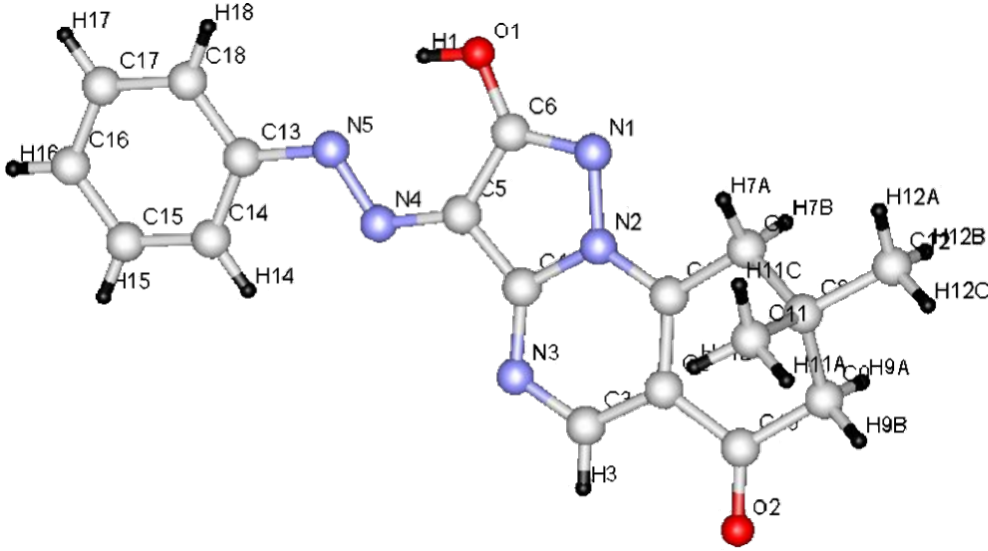
ORTEP diagram of compound **6g**.

Compound **6g** was also obtained by an alternative route: Compound **8** was prepared by reacting enaminone **7** with 5-aminopyrazole derivative **1f** in DMF under microwave heating at 150 °C for 2 min (Table 1). When this compound was refluxed in DMF under microwave heating for 13 min it underwent cyclization to give **6g** (Scheme 1). Moreover, the structure of compounds **6b**–**g** was unequivocally established by single-crystal X-ray diffraction of compounds **6e**,**g** (Figure 3, Figure 4 and Table 3, Table 4) [27–28].

**Table 4:** Selected bond lengths and bond angles for compound **6g**.

bond lengths		bond angles	

atoms numbers	geometric parameter (Å)	atom numbers	geometric parameter (°)

N3–C4 N3–C3 N1–C6 N2–C4 N2–C1 C1–C7 C1–C2	1.330 (2) 1.321(19) 1.343 (17) 1.393 (18) 1.343 (19) 1.491 (2) 1.394 (2)	C3–N3–C4 N1–N2–C1 C1–N2–C4 N2–C1–C2 C4–C5–C6 C1–C2–C10 N3–C3–C2 N3–C4–C5 N2–N1–C6 N1–N2–C4 N2–C4–N3	116.18 (10) 124.04 (12) 121.41 (12) 116.52 (13) 105.52 (13) 119.58 (13) 123.90 (14) 132.56 (14) 104.27 (11) 114.50(11) 123.02 (13)

**Scheme 2:**
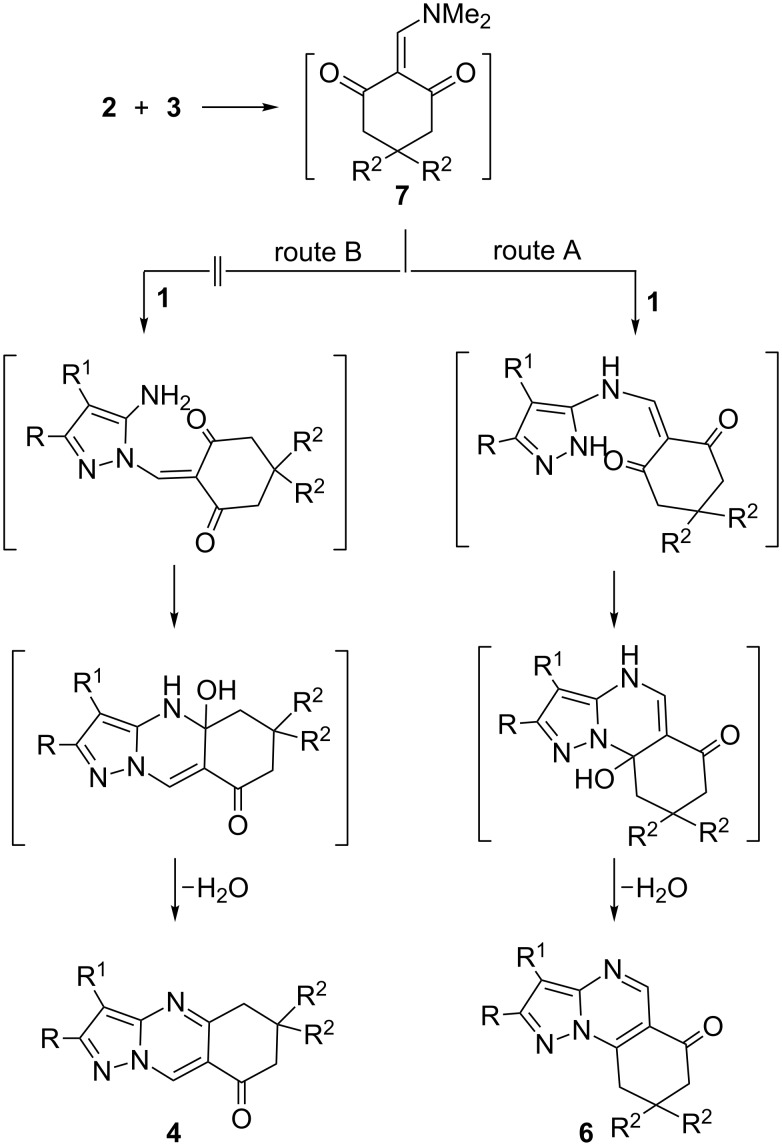
A proposed mechanism to account for the formation of products **6**. The factors that determine the nature of the end product are, however, at present unclear.

A proposed mechanism to account for the formation of products **6** is illustrated in Scheme 2. The base-catalyzed reaction of cyclic 1,3-diketones **2** with DMFDMA **3** gave the enaminone **7**, which subsequently reacted with 5-aminopyrazole **1** at the exocyclic amino function, followed by cyclization through water loss to give **6** (route A). Formation of isomeric product **4**, which would be formed by route B, was ruled out based on spectral and X-ray diffraction data.

From the data of the X-ray crystal structure it can be concluded that the bridged head nitrogen has bond angles closer to those of sp^3^ nitrogen. One may thus conclude that the lone pair on this nitrogen atom does not contribute much to the actual state of the molecule and that charge-separated ions also do not contribute significantly; although, the pyrazolo[5,1-*b*]quinazolin ring is almost planar.

## Conclusion

In summary, we can reveal that the reaction of substituted 5-aminopyrazoles, cyclic 1,3-diketones and dimethyformamide dimethylacetal (DMFDMA, **3**) proceeds by initial attack of the exocyclic amino function. Although an attack by the ring nitrogen has been proposed for the reaction of 5-aminopyrazoles with acrylonitrile [29], here steric factors hinder such an attack and the reaction occurs exclusively, in every case studied, at the amino function.

## Experimental

**General information**. All the reactions were carried out in a Milestone START Microwave Labstation (temperature control by IR sensor). ^1^H NMR (400 MHz) and ^13^C NMR (100 MHz) spectra were measured on a Bruker DPX instrument by using DMSO-*d*_6_ as solvent and TMS as internal standard. Chemical shifts are expressed as δ in ppm. Coupling constants (*J*) are given in Hertz (Hz). The melting points were measured in a Gallenkamp melting-point apparatus and are not corrected. Mass spectra were measured by using VG Autospec Q MS 30 and MS 9 (AEI) spectrometer with the EI (70 eV) mode.

### General procedure for the synthesis of pyrazoloquinazolinones (6a–g)

A solution of 5-aminopyrazole derivative **1a**–**f** (1 mmol), cyclic 1,3-diketones (**2a**,**b**) (1 mmol) and dimethylformamide dimethylacetal (DMFDMA, **3**) (1 mmol) in DMF (10 mL) was heated under reflux in a Milestone Microwave Labstation at 150 °C for 15 min. After concentration and cooling to room temperature, the resulting solid product so formed was collected by filtration, washed well with EtOH, dried and recrystallized from EtOH.

**2,8,8-Trimethyl-8,9-dihydropyrazolo[5,1-*b*]quinazolin-6(7*H*)-one (6a)**: Greenish yellow plates, 201 mg (88% yield); mp 134–135 °C; ^1^H NMR (400 MHz, DMSO-*d*_6_) δ 1.12 (s, 6H, 2CH_3_), 2.48 (s, 3H, CH_3_), 2.56 (s, 2H, CH_2_ at C-9), 3.32 (s, 2H, CH_2_ at C-7), 6.70 (s, 1H, CH at C-3), 8.75 (s, 1H, CH at C-5); ^13^C NMR (100 MHz, DMSO-*d*_6_) δ 14.55, 27.89, 32.36, 36.46, 38.87, 50.08, 98.04, 112.39, 146.03, 149.34, 152.21, 157.52, 194.82; EIMS *m*/*z*: 229.1 (M^+^), 214, 173, calcd. for C_13_H_15_N_3_O 229.28; Anal. calcd for C_13_H_15_N_3_O: C, 68.1; H, 6.59; N, 18.33; found: C, 68.22; H, 6.62; N, 18.35%.

**2-Methyl-8,9-dihydropyrazolo[5,1-*b*]quinazolin-6(7*H*)-one (6b)**: Yellow plates, 170 mg (85% yield); mp 154–155 °C; ^1^H NMR (400 MHz, DMSO-*d*_6_) δ 2.21–2.27 (m, 2H, CH_2_ at C-8), 2.66 (t, *J* = 6.8 Hz, 2H, CH_2_ at C-9), 3.40 (t, *J* = 6.4 Hz, 2H, CH_2_ at C-7), 6.71 (s,1H, CH at C-3), 8.77 (s, 1H, CH at C-5); ^13^C NMR (100 MHz, DMSO-*d*_6_) δ 14.53, 19.95, 33.37, 36.54, 97.91, 113.3, 146.3, 149.0, 153.9, 157.42, 194.81; EIMS *m*/*z* 201.12 (M^+^), calcd for C_11_H_11_N_3_O 201.22; Anal. calcd for C_11_H_11_N_3_O: C, 65.66; H, 5.51; N, 20.88; found: C, 65.68; H, 5.49; N, 20.67%.

**Ethyl 2-amino-6-oxo-6,7,8,9-tetrahydropyrazolo[5,1-*b*]quinazolin-3-carboxylate (6c):** Yellow crystals, 243 mg (89% yield); mp 184–185 °C; ^1^H NMR (400 MHz, DMSO-*d*_6_) δ 1.31 (t, *J* = 7.2 Hz, 3H, CH_3_), 2.10–2.20 (m, 2H, CH_2_ at C-8), 2.63 (t, *J* = 6.8 Hz, 2H, CH_2_ at C-9), 3.25 (t, *J* = 6.8 Hz, 2H, CH_2_ at C-7), 4.31 (q, *J* = 6.8 Hz, 2H, CH_2_), 6.7 (br s, 2H, NH_2_), 8.82 (s, 1H, CH at C-5); EIMS *m*/*z* 274.1 (M^+^), 228, 174.1, calcd for C_13_H_14_N_4_O_3_ 274.28; Anal. calcd for C_13_H_14_N_4_O_3_: C, 56.93; H, 5.14; 20.43; found: C, 57.12; H, 5.23; N, 20.45%

**2,8,8-Trimethyl-3-phenyl-8,9-dihydropyrazolo[5,1-*b*]quinazolin-6(7*H*)-one (6d):** Pale yellow crystals, 253 mg (83% yield); mp 279–280 °C; ^1^H NMR (400 MHz, DMSO-*d*_6_) δ 1.15 (s, 6H, 2 CH_3_), 2.49 (s, 2H, CH_2_ at C-9), 2.58 (s, 3H, CH_3_ at C-2), 2.63 (s, 2H, CH_2_ at C-7), 7.13–7.55 (m, 5H, Ph-H), 8.83 (s, 1H, CH at C-5); ^13^C NMR (100 MHz, DMSO-*d*_6_) δ 14.41, 24.42, 27.90, 36.42, 38.87, 50.15, 112.99, 119.22, 125.88, 126.67, 128.30, 129.20, 132.43, 140.64, 144.52, 159.05, 194.70; EIMS *m*/*z* 305.2 (M^+^), 299, 179.1, calcd for C_19_H_19_N_3_O 305.37; Anal. calcd for C_19_H_19_N_3_O: C, 74.73; H, 6.27; N, 13.76; found: C, 74.66; H, 6.35, N, 13.82%.

**2-Phenyl-8,9-dihydropyrazolo[1,5-*a*]quinazolin-6(7*H*)-one (6e):** Pale yellow crystals, 215 mg (82% yield); mp 197–198 °C; ^1^H NMR (400 MHz, DMSO-*d*_6_) δ 2.25 (m, 2H, CH_2_ at C-8), 2.64 (t, *J* = 5.6 Hz, 2H, CH_2_ at C-9), 3.41 (t, *J* = 5.6 Hz, 2H, CH_2_ at C-7), 7.39 (br s, 1H, CH at C-3), 7.48 (m, 3H, Ph-H), 8.08 (d, *J* = 7.2 Hz, 2H, Ph-H), 8.78 (s, 1H, CH at C-5); ^13^C NMR (100 MHz, DMSO-*d*_6_) δ 19.97, 23.46, 36.63, 79.19, 95.49, 114.10, 126.44, 129.0, 129.69, 131.85, 146.77, 149.69, 154.39, 157.60, 162.32, 194.84; EIMS *m*/*z* 263.1 (M^+^), 235.1, 152.1, calcd. for C_16_H_13_N_3_O 263.11; Anal. calcd for C_16_H_13_N_3_O: C, 72.99; H, 4.98; N, 15.96; found: C, 72.94; H, 5.18; N, 16.32%.

**8,8-Dimethyl-2-phenyl-8,9-dihydropyrazolo[1,5-*a*]quinazolin-6(7*H*)-one (6f):** Pale yellow crystals, 242 mg (83% yield); mp 244–245 °C; ^1^H NMR (400 MHz, DMSO-*d*_6_) δ 1.18 (s, 6H, 2 CH_3_), 2.59 (s, 2H, CH_2_ at C-9), 3.44 (s, 2H, CH_2_ at C-7), 7.34 (s, 1H, CH at C-3), 7.50 (m, 3H, Ph-H), 8.09 (m, 2H, Ph-H), 8.81 (s, 1H, CH at C-5); ^13^C NMR (100 MHz, DMSO-*d*_6_) δ 28.47, 32.73, 37.17, 50.86, 95.94, 113.79, 127.02, 129.29, 129.97, 132.53, 146.90, 150.61, 152.87, 158.37, 194.85; Anal. calcd for C_18_H_17_N_3_O: C, 74.20; H, 5.88; N, 14.42; found: C, 74.32; H, 5.91; N, 14.44%.

**2-Hydroxy-8,8-dimethyl-3-(phenyldiazenyl)-8,9-dihydropyrazolo[1,5-*a*]quina-zolin-6(7*H*)-one (6g)**: Orange crystals, 295 mg (88% yield); mp 254–255 °C; ^1^H NMR (400 MHz, DMSO-*d*_6_) δ 1.14 (s, 6H, 2 CH_3_), 2.66 (s, 2H, CH_2_ at C-9), 3.26 (s, 2H, CH_2_ at C-7), 7.45 (t, *J* = 7.2 Hz, 1H, Ph-H), 7.55 (t, *J* = 7.6 Hz, 2H, Ph-H), 7.85 (d, *J* = 7.6 Hz, 2H, Ph-H), 8.95 (s, 1H, CH at C-5); ^13^C NMR (100 MHz, DMSO-*d*_6_) δ 27.96, 32.25, 36.44, 50.14, 79.20, 115.14, 115.74, 121.33, 129.34, 129.80, 144.26, 148.99, 151.95, 152.61, 162.10, 194.3; EIMS *m*/*z* 335.1 (M^+^), 307.1, 258.1, calcd for C_18_H_17_N_5_O_2_ 335.14; Anal. calcd for C_18_H_17_N_5_O_2_: C, 64.47; 5.11; 20.88; found: C, 64.43; 5.33; 20.95%.

### Synthesis of (*Z*)-5,5-dimethyl-3-[(3-methyl-1*H*-pyrazol-5-yl)amino]cyclohexanone (**5**)

A solution of **1a** (1 mmol) and **2a** (1 mmol) in DMF (10 mL) was heated under reflux in a Milestone Microwave Labstation at 150 °C for 10 min. After concentration and cooling to room temperature, the resulting solid product so formed was collected by filtration, washed well with EtOH, dried and recrystallized from EtOH to afford a pure sample of compound **5** as yellow crystals, 186 mg (85% yield); mp 233–235 °C.

**Synthesis of 4a**: A solution of **1a** (1 mmol) and **2a** (1 mmol) in DMF (10 mL) was heated under reflux in a Milestone Microwave Labstation at 150 °C for 10 min. After concentration and cooling to room temperature, the resulting solid product so formed was collected by filtration, washed well with EtOH, dried and recrystallized from EtOH to afford a pure sample of (*Z*)-3,3-dimethyl-5-(3-methyl-1*H*-pyrazol-5-ylimino)cyclo-hexanone (**5**) as yellow crystals, 186 mg (85% yield); mp 233–235 °C.

**Reaction of 5 with dimethylformamide dimethylacetal (DMFDMA, 3)**: A solution of **5** (1 mmol) and DMFDMA (**3**) (1 mmol) in DMF (10 mL) was heated under reflux in a Milestone Microwave Labstation at 150 °C for 10 min. After evaporation to dryness under reduced pressure, the resulting solid product was collected by filtration, washed well with EtOH, dried and recrystallized from EtOH to give **4a**.

**Alternative synthesis of 6g: Synthesis of 2-((3-hydroxy-4-(phenyldiazenyl)-1*H*-pyrazol-5-ylamino)methylene)-5,5-dimethylcyclohexane-1,3-dione (8):** A solution of **1f** (1 mmol), enaminone **7** (1 mmol) in DMF (10 mL) was heated under reflux in a Milestone Microwave Labstation at 150 °C for 2 min. After concentration and cooling to room temperature, the precipitated product was collected by filtration, washed well with EtOH, dried and recrystallized from EtOH to give a pure sample of **8** as orange crystals, 303 mg (88% yield); mp 255–256 °C; ^1^H NMR (400 MHz, DMSO-*d*_6_) δ 1.01 (s, 6H, 2 CH_3_), 2.40 (s, 2H, CH_2_), 3.26 (s, 2H, CH_2_), 7.24–7.85 (m, 6H, 5 Ph-H and C*H*-NH), 11.76 (s, 1H, NH), 12.59 (s, 1H, pyrazole NH); ^13^C NMR (100 MHz, DMSO-*d*_6_) δ 27.95, 30.70, 50.12, 109.66, 115.16, 115.74, 121.31, 126.16, 129.32, 129.64, 129.80, 144.34, 148.97, 152.57, 158.40, 194.23, 195.33; EIMS *m*/*z* 353.2 (M^+^), 335.1, 242.1, calcd. for C_18_H_19_N_5_O_3_ 353.15; Anal. calcd for C_18_H_19_N_5_O_3_: C, 61.18; H, 5.42; N, 19.82; found: C, 61.23; H, 5.45; N, 19.92%.

**Cyclization of 8**. A solution of **8** (1 mmol) in DMF (10 ml) was heated under reflux in a Milestone Microwave Labstation at 150 °C for 13 min. The reaction mixture was evaporated to dryness in vacuo. The precipitated solid product was filtered off, washed with a small amount of EtOH, dried and recrystallized from EtOH to give an analytical pure sample of **6g** (identical with an authentic sample, MS, ^1^H NMR and ^13^C NMR).

## References

[1] Kessler M, Baudry M, Lynch G (1989). Brain Res.

[2] McQuaid L A, Smith E C R, South K K, Mitch C H, Schoepp D D, True R A, Calligaro D O, O’Malley P J, Lodge D, Ornstein P L (1992). J Med Chem.

[3] Orvieto F, Branca D, Giomini C, Jones P, Koch U, Ontoria J M, Palumbi M C, Rowley M, Toniatti C, Muraglia E (2009). Bioorg Med Chem Lett.

[4] Watson C Y, Whish W J D, Threadgill M D (1998). Bioorg Med Chem.

[5] Weber L, Illgen K, Almstetter M (1999). Synlett.

[6] Kantevari S, Vuppalapati S V N, Nagarapu L (2007). Catal Commun.

[7] Sami S, Nandi G C, Kumar R, Singh M S (2009). Tetrahedron Lett.

[8] Zhu J, Bienaymé H (2005). Multicomponent Reactions.

[9] Dömling A, Ugi I (2000). Angew Chem, Int Ed.

[10] Simon C, Constantieux T, Rodriguez J (2004). Eur J Org Chem.

[11] Carey F A, Sundberg R J (2007). Advanced Organic Chemistry, Part A.

[12] Erkkilä A, Majander I, Pihko P M (2007). Chem Rev.

[13] Laschat S, Becheanu A, Bell T, Baro A (2005). Synlett.

[14] Kappe C O (2004). Angew Chem, Int Ed.

[15] de la Hoz A, Díaz-Ortiz Á, Moreno A (2005). Chem Soc Rev.

[16] Bonrath W, Paz Schmidt R A, Atta-ur-Rahman (2005). Ultrasound in Synthetic Organic Chemistry. Advances in Organic Synthesis.

[17] Gravotto G, Cintas P J (2006). Chem Soc Rev.

[18] Quiroga J, Mejía D, Insuasty B, Abonía R, Nogueras M, Sánchez A, Cobo J, Low J N (2001). Tetrahedron.

[19] Chebanov V A, Saraev V E, Desenko S M, Chernenko V N, Shishkina S V, Shishkin O V, Kobzar K M, Kappe C O (2007). Org Lett.

[20] Chebanov V A, Saraev V E, Desenko S M, Chernenko V N, Knyazeva I V, Groth U, Glasnov T N, Kappe C O (2008). J Org Chem.

[21] Ghotekar B K, Jachak M N, Toche R B (2009). J Heterocycl Chem.

[22] Sadek K U, Shaker R M, Abd Elrady M, Elnagdi M H (2010). Tetrahedron Lett.

[23] Abd El Latif F M, Barsy M A, Aref A M, Sadek K U (2002). Green Chem.

[24] Mekheimer R A, Sadek K U (2009). J Heterocycl Chem.

[R25] 25CCDC 825123 contains the supplementary crystallographic data for compound **6a**. These data can be obtained free of charge from the Cambridge Crystallographic Data Centre via http://www.ccdc.cam.ac.uk

[R26] 26CCDC 833076 contains the supplementary crystallographic data for compound **5**. These data can be obtained free of charge from the Cambridge Crystallographic Data Centre via http://www.ccdc.cam.ac.uk

[R27] 27CCDC 827653 contains the supplementary crystallographic data for compound **6e**. These data can be obtained free of charge from the Cambridge Crystallographic Data Centre via http://www.ccdc.cam.ac.uk

[R28] 28CCDC 826742 contains the supplementary crystallographic data for compound **6g**. These data can be obtained free of charge from the Cambridge Crystallographic Data Centre via http://www.ccdc.cam.ac.uk

[29] Elnagdi M H (1974). Tetrahedron.

